# The Book World of Medicine and Science

**Published:** 1911-08-12

**Authors:** 


### THE BOOK WORLD OF MEDICINE AND SCIENCE.

The Pupil Midwives' Register of Cases. Arranged by Gertrude Marks. (London: Baillière, Tindall, and Cox. 1910. Price 1s. 6d. net.)

This excellently arranged register is designed to afford space for the complete charting, and all other records, of twenty obstetric cases from start to finish. On the first sheet of each case report are to be entered in appropriate spaces all such details as the name, age, and address of the patient, previous obstetric history, and all particulars of the pregnancy up to labour. Then comes a sheet for the labour report, followed by a chart for the first ten days of the puerperium to show all the usual features, including the involution of the uterus; and following that pages for the daily report to the doctor on the usual lines. Prefixed to these are a few pages of hints as to the questions which are important to ask about the pregnancy, labour, progress of mother and infant, etc.; in fact, a scheme for the anamnesis of a puerperal case. Two minor criticisms may perhaps be offered. One is that the large space left for the description in each labour report of the baby at birth might with advantage be curtailed in order to give more room for the details of labour and delivery. The other, that a specimen case reported right through might prove helpful to those whose obstetric experience is not very great.

Monthly Bulletin of Municipal Statistics of the City of Buenos Aires (for May, June, and July 1910).

These pamphlets, published in English, give an account of the meteorology and hygiene of the city, as well as figures relative to the shipping movements of immigration and emigration, hospital and asylum statistics, inmates of night shelters, births, marriages and deaths, slaughter-houses and sanitary inspection, prisons and police statistics, political economy, including buying and selling of mortgages and movements on the Stock Exchange, the municipal Mont de Piété and Savings Bank, public and private lighting, water supply, education, building, telegraphs, telephones, race-meetings, zoological gardens, postal returns, tramways, railways, and banknotes in circulation. Each issue comprises six pages of closely-printed matter, each sheet being of large foolscap size.

Minding the Baby. A Manual of Infant Care and Management. By Mrs. Leonard Hill. (London: Edward Arnold. 1911. Price 3d. net paper; 6d. net cloth.)

This short volume is intended for the use of teachers in public elementary schools as a class-book for elder girls, as well as for young mothers and those attending or delivering lectures on the care and nurture of infants. For these purposes it seems to us in every way suitable. We have one criticism to offer, and that is that the author is not sufficiently severe on the use of a comforter or dummy. Some medical authorities attribute to its use deformities of the palate in after life and the formation of adenoids. We should like to see it banished entirely from the nursery, as there really is no necessity for its survival. If the child must at any cost have something to suck, let it be a large bone-ring, which can easily be kept clean, and which it cannot suck in the same way as if it were drawing in food. Such a ring does not perish in the way rubber comforters do, and consequently does not collect as much dirt. Most babies properly brought up will not need much even of the ring to keep them quiet. Another small point; the author very properly tells the mother never to try soothing-powders, but she omits to state the reason why these should never be given. It might make her lesson more impressive if she were to state that nine out of ten of the so-called "soothing" preparations contain opium in one form or another. Some unfortunate infants have been "soothed" to such purpose that the only trouble they have caused afterwards has been to force their mothers to attend a coroner's inquest and subsequently a funeral.

An Introduction to Surgery. By Rutherford Morison, M.A., M.B., F.R.C.S. (Bristol: John Wright and Sons, Ltd. Price 8s. 6d. net. Interleaved, 9s. 6d. net.)

The matter embodied in this book is an abstract from the author's lectures, and the aim of the work is to emphasise the general principles of surgery in regard to diagnosis and treatment, based on a sound knowledge of pathology. It is more therefore an aid to clinical study than—as is the case with most surgical text-books—a treatise on surgery. Read in this light, it is really a most excellent work. If the beginner is to understand to any advantage the science of surgery, he must get a good grasp of the general principles which underlie that subject, and until those general principles are thoroughly mastered he can neither appreciate nor understand the clinical cases he meets with in the wards, nor the autopsies he may see in the post-mortem room. At the outset of his clinical study the student is presented with a mass of detail of which he does not understand the why and wherefore, and the grasping of which seem to him surrounded by insurmountable difficulties. But directly he begins to get a grasp of the general principles of surgery, as the author rightly says "to understand the value of their universal application in so-called 'special' regions, that is to disease affecting any portion of the body," he ceases to be overcome by awe at the apparently enormous amount he has to learn, and the light of reason begins to dawn in the dark places of his mind. and he begins to think and reason out things for himself. What before had appeared absolutely perplexing now appears rational. And the mission of this book is undoubtedly to teach the student of surgery upon these lines, and to our mind it is indeed an excellent method and one which we should like to see more often followed. For the book we have nothing but praise, the get-up is excellent, the letterpress is good, and the illustrations are amongst the finest we have seen, most of them being from actual photographs. One of the best chapters is on gangrene. The various forms of gangrene are fully described and are divided into two groups, predisposing and exciting, and each group is treated and described in detail. We notice with satisfaction that the essential difference between septic and clean forms of gangrene are emphasised. "There is no such thing as a genuine dry gangrene. Every ordinary gangrene is originally moist, and whether it becomes dry depends upon whether it can be kept from organismal infection or goes septic. Dry is an aseptic, and moist a septic gangrene." Another excellent chapter is that on syphilis, tubercle, and malignant disease, and there is a good section on indications for operation. At the end of the book there are several excellent skinagrams. The whole work reflects the greatest credit upon the author, and we heartily congratulate him upon producing such a helpful contribution to the library of surgery.

The Prevention of Sexual Diseases. By Victor G. Vecki, M.D. (New York: The Critic and Guide Co. 1910. Pp. 132. Price $1.50.)

It can hardly be gainsaid that more active measures are called for in order to prevent the spread of syphilis and gonorrhœa among the community, and it is not only true that the question is one which has not been handled effectively, but has been obscured by a great deal of cant and hypocrisy. The question, presenting as it does so many difficulties, is one that should not be left to moralists only; it is to be feared that the influence of the medical profession has not made itself felt sufficiently on this subject. Any success from measures directed to the suppression of prostitution is impossible of realisation. The inefficiency of systems of inspection is well known, though in this direction something can certainly be done, and a few, at least, of the dangerous cases be prevented from spreading their disease to ignorant and careless victims. We do not intend to comment in detail on this volume by Dr Vecki, but to indicate the character of the book. He treats the subject vigorously and openly, but unfortunately in a somewhat unsavoury spirit. The book is obviously written for a non-medical public, and in places the melodramatic colouring is thickly laid on. An introduction by Dr. Wm. Robinson is no embellishment to the book, in fact it constitutes a prejudicial blot on account of its immoderate and ludicrous language. Dr. Vecki devotes attention to many sides of the question, including the duty of the Government and the duty of the physician, and also goes into the details of personal prophylaxis. We may cordially agree with the trenchant remarks on the failure of the Press to free its pages from the degrading and pernicious advertisements of the quacks, to whom, perhaps, the major part of the dangers of sexual diseases may be ascribed. While it may be admitted that the dangers and horrors described are matters of fact, we must deprecate the whole tone of the book, which is calculated to exert a pernicious influence under a pretence of beneficial knowledge. The book is fortunately intended for American consumption, and we have every confidence that something better is available on this side of the Atlantic.

Urine Examination made Easy. By Thomas Carruthers, M.A., M.B., Ch.B. Second Edition. (London: J. and A. Churchill. 1911. Pp. 47. Price 1s. 6d. net.)

The knowledge of urine testing possessed by nurses is unfortunately not always of that high order necessary for the care and accuracy which its performance demands, and hence we welcome any measures tending to remedy this defect in their training. Dr. Carruthers has certainly made a praiseworthy effort to reduce urine testing to its simplest formula, and we have not the least doubt that his demonstrations to nurses are models of clearness. In this little book we have a careful and thoughtful explanation of urine testing, though its subdivisions and cross-references appear to us to be a little confusing, and we think that the whole matter might have been made simpler if it had been distributed over a less number of pages. Under the heading "appearances" we note the omission of the common characteristics of diabetic urine, and it would have been as well to have described the composition of Payne's reagent referred to on page 34. In using Fehling's solution the best method is to boil the solution and the urine in separate test-tubes, and then to add the urine to the Fehling's solution while both are hot, when an immediate reaction is to be expected. But we must admit that on the whole these notes fulfil their object very well, and we can cordially recommend the little book to the attention of nurses as the most useful exposition of this very important part of their duties we have come across.

Questions of the Day and of the Fray: No. II. Mental Defect, Mal-nutrition, and the Teacher's Appreciation of Intelligence. A Reply to Criticisms of the Memoir on "The Influence of Defective Physique and Unfavourable Home Environment on the Intelligence of School Children." By David Heron, D.Sc. (London: Dulau and Co., Ltd. 1911. Pp. 34. Price 1s. net.)

This pamphlet contains Dr. Heron's reply to one of his critics, Mr. G. Udney Yule, who wrote in the *Journal of the Royal Statistical Society* and in *School Hygiene*. Dr. Heron seems to have established his case.

